# Supplementary material for: A Compact, Syringe-Assisted, Vacuum-Driven Micropumping Device
Source: Micromachines (Basel). 2019 Aug 17;10(8):543. doi: 10.3390/mi10080543 (PMC6723763; doi:10.3390/mi10080543)
Supplement: Supplementary file 1 [file micromachines-10-00543-s001.zip › Supplemental Materials-578340.docx]

**SUPPLEMENTAL MATERIAL**

A Compact Syringe-Assisted Vacuum-Driven Micropumping Device

Anyang Wang, Domin Koh, Philip Schneider, Evan Breloff and Kwang W. Oh*

The gas pressure inside the vacuum chamber can be described using ideal gas laws. An equivalent pressure model is shown in **Figure S1**.

**Figure S1.** An equivalent model of the gas pressure inside channel.$P_{\mathrm{ATM}} (\mathrm{Pa})$ is atmospheric pressure, ${\Delta P}_{cap1} (\mathrm{Pa})$ is capillary pressure between gas-liquid at inlet, $\Delta P (\mathrm{Pa})$ is pressure difference across the liquid column, ${\Delta P}_{cap2} (\mathrm{Pa})$ is capillary pressure between gas-liquid in channel, ${\Delta P}_{\mathrm{gas}}\left( t \right) (\mathrm{Pa})$ is the gas pressure inside channel, $P_{\mathrm{VAC}} (\mathrm{Pa})$ is vacuum pressure generated by a pump, $V\left( t \right) {(m}^{3}$) is gas volume inside channel, $\Delta N_{A} (\mathrm{mol}{\cdot m}^{-2}\cdot s^{-1}$) is diffusion rate of gas molecule, $w (m)$ is the thickness of PDMS membrane and $p$ (Barrer) is permeability coefficient of PDMS.

The molar flux diffusion, $\Delta N_{A}(mol\cdot m^{-2}\cdot s^{-1})$ out of microfluidic channels into a vacuum chamber can be written using the permeability coefficient$p$,

$\Delta N_{A}=\frac{1}{w}p\left( P_{\mathrm{gas}}\left( t \right)-P_{\mathrm{VAC}} \right)$ (

, where $w (m)$ is the thickness of PDMS membrane, $p (Barrer$) is the permeability coefficient of PDMS, $P_{\mathrm{gas}}\left( t \right) (Pa)$ is the gas pressure inside the channel, $P_{\mathrm{VAC}} (Pa)$ is the pressure inside the vacuum chamber.

The pressure inside the vacuum chamber was calculated using the ideal gas law. The schematics of a syringe pump plunger and a vacuum chamber is shown in **Figure S2**.

**Figure S2.** An equivalent model of the pressure inside the vacuum chamber.

Before pulling the syringe, the ideal gas law for the vacuum chamber and syringe can be written as

$P_{\mathrm{VAC}\_0}V_{\mathrm{VAC}\_0}=n_{\mathrm{VAC}\_0}\mathrm{RT}$ (

$P_{\mathrm{SYR}\_0}V_{\mathrm{SYR}\_0}=n_{\mathrm{SYR}\_0}\mathrm{RT}$ (

, where $P_{VAC\_0} (\mathrm{Pa}), V_{\mathrm{VAC}_{0}}(m^{3})$ is the initial pressure, the volume inside the vacuum chamber respectively and $P_{\mathrm{SYR}_{0}}(\mathrm{Pa}), V_{\mathrm{SYR}_{0}}(m^{3})$ is the initial pressure, the volume of the connected syringe respectively. To be specific, before pulling the syringe, the pressure inside the vacuum chamber is atmospheric pressure, $P_{\mathrm{VAC}\_0}=101 \mathrm{kPa}$. Also, there are no gas molecules inside the syringe before pulling the syringe, $n_{\mathrm{SYR}\_0}=0 \mathrm{mol}.$

After pulling the syringe,

$P_{\mathrm{VAC}}\left( V_{\mathrm{VAC}}+V_{\mathrm{SYR}} \right)={(n}_{\mathrm{VAC}\_0}+n_{\mathrm{SYR}\_0})\mathrm{RT}$ (

Considering $V_{\mathrm{SYR}}(\sim\mathrm{ml})\gg V_{\mathrm{VAC}}(\sim\mu l)$, $n_{\mathrm{SYR}\_0}=0 \mathrm{mol}$,

$P_{\mathrm{VAC}}V_{\mathrm{SYR}}=n_{\mathrm{VAC}\_0}\mathrm{RT}$ (

Therefore,

$P_{\mathrm{VAC}}=P_{\mathrm{VAC}\_0}\times\frac{V_{\mathrm{VAC}\_0}}{V_{\mathrm{SYR}}}$ (

For example, when $V_{VAC\_0}=1.24 \mu l$, $P_{\mathrm{VAC}\_0}=101 \mathrm{kPa},$ $V_{\mathrm{SYR}}=1 \mathrm{ml}$, the pressure inside the vacuum chamber can be calculated to be $P_{\mathrm{VAC}}=124$ Pa.
